# Nationwide Trends in Healthcare Utilization and Expenditures Among Patients with Cervical Dystonia in Korea: A 7-Year Analysis Using Health Insurance Data

**DOI:** 10.3390/healthcare13222995

**Published:** 2025-11-20

**Authors:** Ching-Wen Huang, Bo-Hyoung Jang

**Affiliations:** Department of Preventive Medicine, College of Korean Medicine, Kyung Hee University, Seoul 02447, Republic of Korea

**Keywords:** cervical dystonia, spasmodic torticollis, medical expenditures, national health insurance service

## Abstract

**Background/Objectives:** Cervical dystonia (CD) is the most common focal dystonia, but nationwide evidence on healthcare use is limited. This study assessed trends in utilization and expenditures in Korea. **Methods:** Using National Health Insurance Service claims (2017–2023), we identified CD patients (ICD-10 G24.3) and analyzed annual patients, visits, and expenditures by demographics, medical system, service type, and specialty. **Results:** A total of 6614 patients (33,896 claims) were included. Patient numbers declined until 2021 then slightly rebounded, and total expenditures fluctuated. Women were more prevalent, but men incurred higher costs. Western Medicine (WM) expenditures tended to increase while Korean Medicine (KM) tended to decline. Core botulinum toxin costs remained broadly stable; physiotherapy and diagnostic testing increased, particularly WM outpatient testing. Outpatient injection-related and anesthesia expenditures showed decreasing tendencies, while hospitalization and radiology exhibited modest or minimal changes. Neurology managed the most patients, while neurosurgery generated the highest costs. **Conclusions:** CD care in Korea showed declining patient numbers alongside overall stable total expenditures, with increasing use of rehabilitation and diagnostic services and continued stable use of botulinum toxin as a core therapy.

## 1. Introduction

Cervical dystonia (CD), also known as spasmodic torticollis, is the most common form of focal dystonia in adults. It is characterized by sustained or intermittent contractions of the cervical muscles, leading to abnormal head posture, involuntary movements, chronic pain, and functional limitations. Epidemiological estimates of CD prevalence vary substantially across regions and study methodologies. In Europe, community-based registry studies have reported prevalence rates of approximately 4.9 per 100,000 population [1], whereas a hospital-based study from Finland suggested a prevalence as high as 304 per million adults [2]. Such wide discrepancies highlight ongoing debate regarding methodological differences, underdiagnosis, and potential regional variability in CD burden. Although CD is considered a rare disorder, its impact on quality of life is profound, and the long-term need for clinical care is substantial [3,4].

Currently, intramuscular injection of botulinum toxin type A (BoNT-A) is globally recognized as the first-line treatment for CD. Clinical benefit typically emerges within several days of injection and lasts for approximately three to four months, necessitating repeated treatments throughout the year. Alternative therapeutic approaches include deep brain stimulation, oral pharmacotherapy, physical therapy, and rehabilitation [5]. More recently, emerging interventions such as wearable monitoring devices and adaptive neuromodulation techniques have been introduced, offering additional options for clinical management [6]. However, the relative cost-effectiveness of these diverse approaches remains uncertain.

The economic burden of CD is considerable. Studies have demonstrated that healthcare expenditures among CD patients are substantially higher compared to the general population. For example, U.S. insurance claims data showed that the average annual cost for CD patients was USD 20,168, compared with USD 7141 for matched controls [7]. Similar findings have been observed across European countries, where costs vary by healthcare system but consistently highlight the significant financial impact of CD. Moreover, indirect costs—including loss of productivity, disability, and psychological distress—further contribute to the overall burden [7,8].

In Korea, the healthcare system is structured as a dual-track model, encompassing both Western Medicine (WM) and Korean Medicine (KM). Patients are free to choose between the two systems of care, and both are covered by the National Health Insurance (NHI). However, nationwide studies describing the utilization patterns and economic burden of CD within this dual medical framework remain scarce. Compared with Western countries, where cervical dystonia care is almost exclusively delivered within Western Medicine (WM) settings and typically managed through specialist-based and injection-oriented treatments [7,8], Korea operates a dual medical system that integrates both WM and Korean Medicine (KM) under the National Health Insurance framework. This structure allows patients to seek a broader range of treatment modalities—including pharmacologic, rehabilitative, and traditional approaches—within the same insurance system. As a result, patterns of healthcare utilization in Korea may be more diverse than those observed in Western countries, reflecting the coexistence of conventional and traditional medical practices. The study was conceptually informed by Andersen’s Behavioral Model of Health Services Use, which posits that healthcare utilization is influenced by predisposing (e.g., age, sex) and enabling factors (e.g., accessibility and insurance coverage). To address this gap, the present study analyzes data from the Korean National Health Insurance Service (NHIS) between 2017 and 2023 to evaluate trends in healthcare utilization and expenditures among CD patients. Our findings indicate a decline in patient numbers but overall stable total expenditures with category-level shifts (increased outpatient testing and rehabilitation), with distinct patterns across WM and KM, providing evidence relevant for clinical practice and healthcare policy.

Accordingly, this study aimed to assess nationwide trends in healthcare utilization and expenditures among patients with cervical dystonia between 2017 and 2023. Specifically, we addressed three research questions: (1) How did healthcare utilization and expenditures for cervical dystonia change over time within Korea’s dual medical system (2) Which service categories (e.g., diagnostic testing, rehabilitation) were the major cost drivers? (3) How did utilization and expenditure trends vary across sex and age groups? The remainder of this paper is organized as follows: Section 2 describes the data sources and analytic methods; Section 3 presents the main results; Section 4 discusses the findings in relation to international evidence and policy implications; and Section 5 concludes with limitations and directions for future research.

## 2. Materials and Methods

### 2.1. Data Source

This study utilized customized claims data from the Korean National Health Insurance Service (NHIS), which provides universal health coverage to the entire Korean population. The NHIS database includes both inpatient and outpatient claims and contains comprehensive information on diagnostic codes, procedures, service categories, prescription records, medical expenditures, and patient demographics such as age and sex. In addition, all medical services delivered under both Western Medicine (WM) and Korean Medicine (KM) systems are covered by the National Health Insurance and recorded in the claims database. The study period spanned from 1 January 2017 to 31 December 2023.

De-identified customized claims data were obtained from the National Health Insurance Service (NHIS) through its official data provision process for research use. The research protocol and Institutional Review Board (IRB) approval were submitted to the NHIS Data Provision Review Committee for evaluation. After approval, de-identified data were extracted according to the study specifications and analyzed within the NHIS secure virtual data analysis environment, which has no external network access. Only aggregated results were permitted to be exported after NHIS review. All procedures complied with the NHIS data security and confidentiality regulations.

### 2.2. Study Population

**Figure 1:**
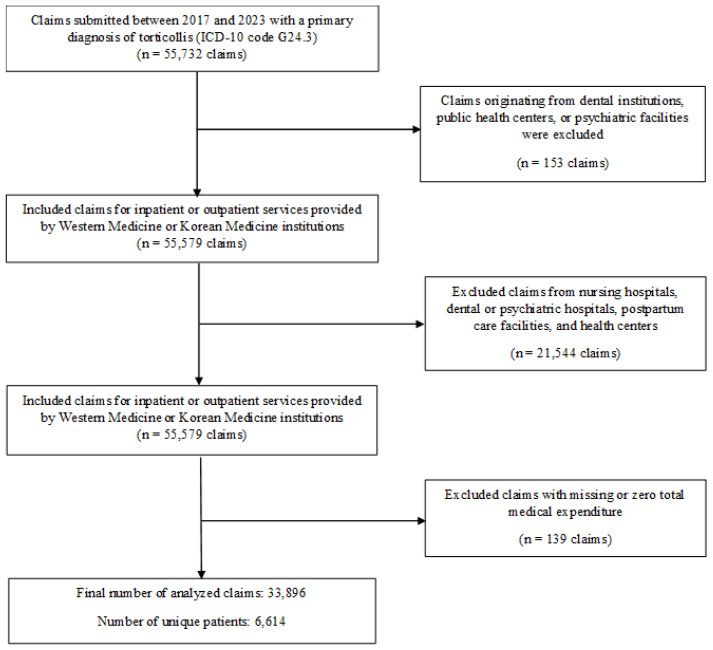
Selection Process of the Study Population.

Patients with a primary diagnosis of cervical dystonia (ICD-10 code G24.3) were identified from NHIS claims submitted during the study period. In the Korean NHIS/HIRA system, the primary diagnosis represents the main reason for care and is coded by the treating physician. CD cases were defined using ICD-10 code G24.3 recorded as the primary diagnosis in inpatient and outpatient claims. Both inpatient and outpatient encounters were included. A total of 55,732 claims were initially identified, and the following exclusion criteria were applied sequentially, as illustrated in Figure 1: claims originating from dental institutions, public health centers, or psychiatric facilities (n = 153); claims submitted by nursing hospitals, psychiatric hospitals, dental hospitals, postpartum care facilities, and health centers (n = 21,544) were also excluded to ensure consistency in care settings, as these institutions predominantly provide long-term residential or non-CD-specific services that are not comparable to acute outpatient or inpatient CD management; and claims with missing or zero total medical expenditure (n = 139). After applying these criteria, the final analytic cohort comprised 33,896 eligible claims, representing 6614 unique patients.

### 2.3. Variables and Measures

The study variables included demographic characteristics (age, categorized in 10-year intervals, and sex). Age groups were categorized in 10-year intervals to ensure sufficient sample size and stable estimates, consistent with prior claims-based studies using Korean NHIS data [9,10,11]. Medical system type (Western Medicine (WM) vs. Korean Medicine (KM), classified according to institutional type). Healthcare utilization indicators comprised the number of unique patients, number of visits, and total reimbursed medical expenditure (in USD). Both total expenditures and averages (per patient and per visit) were calculated to enable comparisons across subgroups and over time. Service categories were analyzed, including consultation fees, hospitalization, medication and prescription costs, injection procedures (including botulinum toxin), physiotherapy, treatment, diagnostic testing, radiology, and rehabilitation, as well as utilization patterns by medical specialty (e.g., neurology, neurosurgery, rehabilitation, and Korean medicine). These service categories were defined according to the claim classification variable in the NHIS database, which categorizes each claim by type of medical service based on the official NHIS fee schedule. All expenditures were converted from KRW to USD at a fixed rate of 1 USD = 1388 KRW; no inflation or PPP adjustments were applied.

### 2.4. Statistical Analysis

Descriptive analyses were conducted to examine healthcare utilization and expenditures by calendar year, sex, age group, and medical system. Temporal trends were visualized using line and area graphs. In addition, simple linear regression analyses were performed to formally assess temporal trends in patient numbers and expenditures from 2017 to 2023, treating year as a continuous variable. The regression coefficient (β) represented the average annual change, and a *p*-value < 0.05 was considered statistically significant. Compound annual growth rates (CAGR) were calculated for key utilization and expenditure indicators to assess longitudinal changes. All statistical analyses and data visualizations were performed using R software (version 4.3.1), with the ggplot2 package applied for graphical presentation.

### 2.5. Ethics

This study was reviewed and approved by the Institutional Review Board of Kyung Hee University (KHSIRB-24-661(EA)). The requirement for informed consent was waived due to the use of de-identified administrative data.

## 3. Results

### 3.1. Overall Trends in Healthcare Utilization of CD Patients

**Figure 2:**
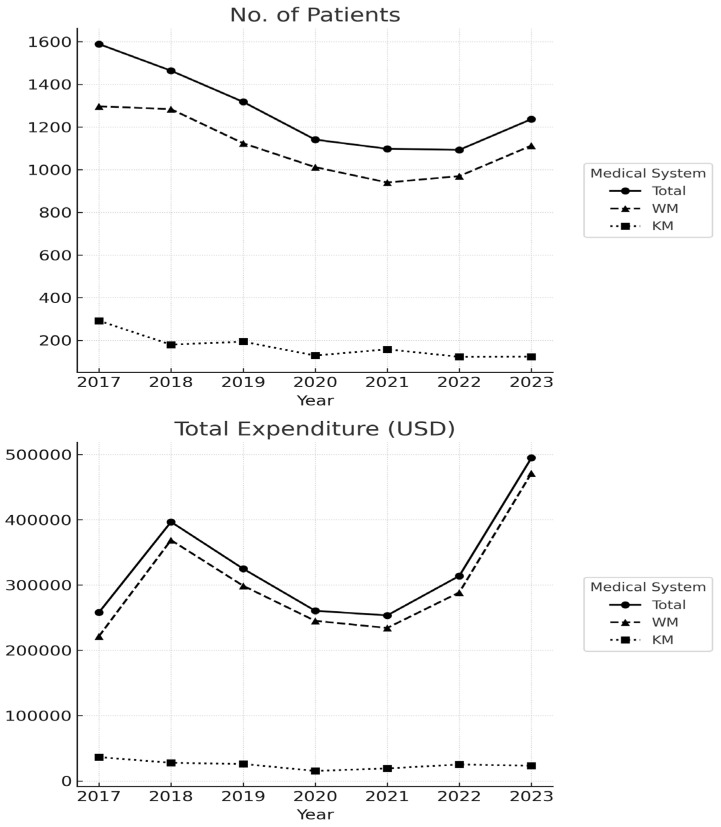
Annual Trends in Patient Numbers and Healthcare Expenditures.

Between 2017 and 2023, the overall utilization patterns for cervical dystonia (CD) care exhibited a gradual decline in patient numbers alongside fluctuating expenditures (Figure 2). Linear regression analysis (Table 1) confirmed a statistically significant annual decrease in the total number of CD patients (β = −72.1, *p* = 0.026, R^2^ = 0.66). In contrast, total and Western Medicine (WM) expenditures showed modest but statistically non-significant increases, whereas Korean Medicine (KM) expenditures displayed a non-significant downward tendency. These results indicate that although the number of patients decreased over time, the average expenditure per patient showed an upward tendency, particularly within the Western Medicine (WM) system.

The annual counts and expenditure values used for this analysis are provided in Supplementary Table S1, which shows a steady reduction in CD patients until 2021 followed by a small numerical uptick through 2023. Expenditure fluctuations—including an increase in 2018, a temporary decline during 2020–2021, and a rebound in 2023—were primarily driven by the WM sector, whereas KM utilization and costs remained consistently low throughout the study period.

### 3.2. Distribution of Patients by Sex and Age Group

Across all study years, the number of patients among female patients (n = 2613) exceeded that of male patients (n = 2229). However, the average annual expenditure was higher for males (USD 176,383) than for females (USD 152,543). Average per-patient expenditure was also higher in men across both medical systems, suggesting that the greater total cost for males reflects moderately higher intensity of care rather than patient volume alone. The compound annual growth rate (CAGR) of total healthcare expenditures for male patients was positive in the WM system (+10.9%) but negative in the KM system (−6.4%). For female patients, expenditures demonstrated a numerical increase in WM (+15.4%) but declined in KM (−7.7%). Linear regression analysis further supported these temporal patterns. A significant downward trend in the annual number of patients was observed in WM males (β = −39.25, 95% CI −54.42 to −24.08, *p* < 0.001) and in both KM males (β = −14.11, 95% CI −18.85 to −2.43, *p* = 0.02) and KM females (β = −12.71, 95% CI −22.26 to −3.17, *p* = 0.02), whereas WM females showed no significant change over time (*p* = 0.54). In terms of expenditure, no significant annual changes were detected in any group (all *p* > 0.05). (Table 2).

Age distribution revealed that patients aged 40–69 years accounted for the largest share of healthcare use. Within the WM system, the largest CAGR values were observed in the 40–49 age group (CAGR +31.9%) and the 70–79 age group (+31.6%), followed by the ≥80 age group (+20.0%), indicating higher utilization levels among middle-aged and older populations. In the KM system, the highest CAGR values were noted in the ≥80 age group (CAGR +23.0%) and the 70–79 age group (+21.2%), suggesting increasing reliance on KM among the oldest patient groups despite overall declines in younger cohorts. Linear regression analysis confirmed these age-specific trends. In the WM system, significant annual decreases in patient numbers were observed among those aged 0–9 (β = −21, 95% CI −30 to −13, *p* < 0.001), 10–19 (β = −6, 95% CI −9 to −3, *p* = 0.01), 20–29 (β = −5, 95% CI −8 to −2, *p* = 0.01), and 40–49 years (β = −8, 95% CI −13 to −3, *p* = 0.01), whereas older age groups (≥50 years) showed no significant changes (*p* > 0.05). Regarding expenditures, significant annual decreases were also identified among the youngest WM patients aged 0–9 (β Exp = −665 USD, 95% CI −1329 to −0.3, *p* = 0.05) and 20–29 years (β Exp = −1531 USD, 95% CI −3031 to −30, *p* = 0.05), based on a conversion rate of 1 USD = 1388 KRW. In the KM system, expenditures also declined significantly among younger groups, including 10–19 years (β Exp = −46 USD, 95% CI −80 to −11, *p* = 0.02) and 20–29 years (β Exp = −829 USD, 95% CI −1363 to −294, *p* = 0.01), while other age groups showed no significant changes (*p* > 0.05). In contrast, younger age groups (10–29 years) showed notable numerical declines in utilization, with CAGRs ranging from −13.3% to −42.0% across both WM and KM systems. (Table 3).

**Figure 3:**
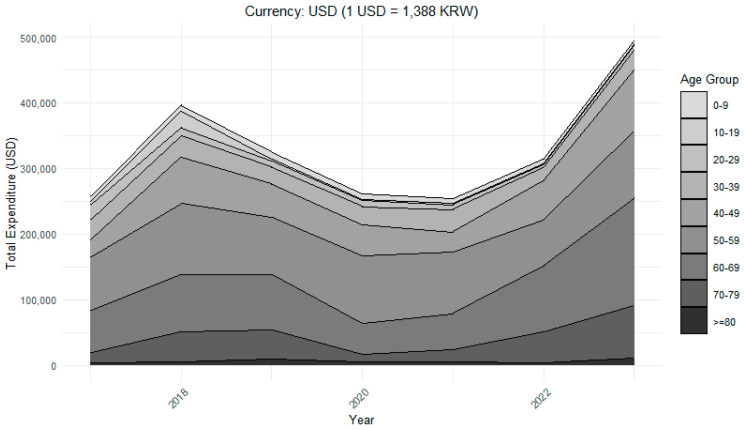
Total Healthcare Expenditures by Age Group.

As illustrated in Figure 3, middle-aged patients (40–69 years) accounted for the largest share of total expenditures throughout the study period, while elderly groups (≥70 years) showed a relative increase in spending after 2020. In contrast, younger age groups (<30 years) contributed minimally to overall expenditures.

### 3.3. Expenditure by Type of Medical Service

As shown in Table 4, consultation fees represented the largest category of expenditures within the WM outpatient setting (3521 visits annually), although utilization remained relatively stable, showing only a slight and statistically non-significant decline over time (CAGR −0.3%, *p* = 0.09). In the WM inpatient setting, consultation expenditures also showed minimal variation (CAGR +1.6%, *p* = 0.14). Hospitalization expenditures in WM showed a numerical decrease (CAGR −2.9%, *p* = 0.76), in contrast to earlier periods when inpatient costs had been rising.

Patterns varied across service types. Injection-related expenditures showed divergence between settings, with inpatient services increasing slightly (CAGR +2.9%), while outpatient expenditures declined (CAGR −5.0%); the latter trend was statistically significant (*p* = 0.01). In the NHIS claims structure, botulinum toxin injections are included in the ‘Injection fee’ category; therefore, changes in injection-related expenditures or visit counts can indirectly reflect trends in BoNT administration for cervical dystonia. Anesthesia expenditures similarly showed a significant downward trend (CAGR −13.2%, *p* = 0.01). Physiotherapy expenditures rose consistently in both inpatient (CAGR +8.9%, *p* = 0.10) and outpatient (CAGR +6.6%, *p* = 0.58) settings, whereas treatment expenditures were largely stable for inpatients (CAGR 0.0%, *p* = 0.61) but decreased for outpatients (CAGR −10.2%, *p* = 0.63). Diagnostic testing demonstrated growth in the WM outpatient setting (CAGR +11.1%, *p* < 0.001), while remaining nearly unchanged for inpatients (+1.7%, *p* = 0.34). By contrast, radiology expenditures remained stable for inpatients (CAGR +0.7%, *p* = 0.20) but declined numerically in outpatients (CAGR −12.0%, *p* = 0.61).

In the KM system, overall expenditures tended to be lower over time, with most changes not reaching statistical significance, particularly for outpatient consultation (CAGR −12.2%, *p* = 0.73), treatment (CAGR −12.1%, *p* = 0.53), and diagnostic testing (CAGR −40.5%, *p* = 0.74). Nevertheless, certain inpatient categories such as consultation (CAGR +23.2%, *p* = 0.41) and treatment (CAGR +5.8%, *p* = 0.99) showed numerical increases without statistical significance, suggesting a redistribution of resource use within KM services. Outpatient medication expenditures also showed positive growth (CAGR +16.2%, *p* = 0.12), in contrast to the overall downward trend in other KM categories. Overall, most changes across service categories were small in magnitude and statistically non-significant, indicating relative stability in utilization over time.

### 3.4. Distribution of Visits by Medical Department

**Figure 4:**
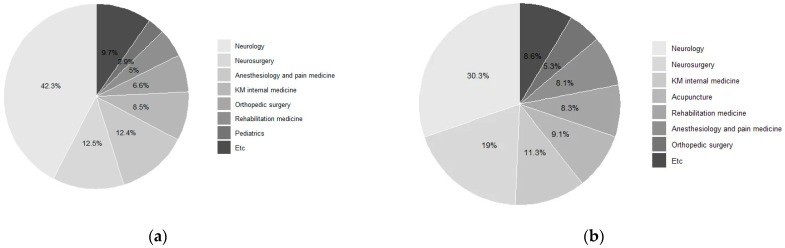
Medical Specialties for Cervical Dystonia: (**a**) Number of Patients; (**b**) Number of Claims.

As shown in Table 5 and Figure 4, neurology accounted for the largest number of patients (n = 2981) and visits (10,282), with total expenditures of USD 504,908. Neurosurgery involved 554 patients and 6434 visits, generating the highest expenditure among all specialties (USD 1,295,531) and the highest average cost per patient (USD 2338) as well as per visit (USD 201).Other frequently utilized specialties included anesthesiology and pain medicine (874 patients, 2741 visits, USD 110,414), rehabilitation medicine (344 patients, 2815 visits, USD 117,910), and orthopedic surgery (558 patients, 1788 visits, USD 42,189).

KM specialties were also represented, including internal medicine (616 patients, 3842 visits, USD 77,026) and acupuncture (121 patients, 3082 visits, USD 61,757), both of which showed relatively low average costs per visit (USD 20) but moderate per-patient expenditures (USD 125 and USD 510, respectively). Additional contributions were observed from pediatrics and adolescents (259 patients, 404 visits, USD 9060), neuropsychiatry (206 patients, 1327 visits, USD 23,401), and general surgery (182 patients, 419 visits, USD 27,796).

## 4. Discussion

### 4.1. Overall Trends in Healthcare Utilization

In this nationwide claims-based analysis, we observed a steady decline in the number of patients with cervical dystonia (CD) from 2017 through 2020, followed by a modest uptick in 2022–2023. This downward trend was statistically significant in linear regression analysis (β = −72.1, *p* = 0.026; Table 1), confirming a consistent temporal decrease across the study period. A similar pattern was also observed in disease statistics from the Korean Health Insurance Review and Assessment Service (HIRA), which reported comparable reductions in CD-related patients between 2018 and 2020. While the reasons behind this trend remain unclear, existing studies have not yet provided a consistent explanation, indicating that further investigation is warranted. It is also possible that the observed decline partly reflects demographic changes or population aging rather than a true reduction in disease burden.

The additional decline during 2020–2022 is consistent with nationwide utilization drops during the COVID-19 pandemic, when reduced accessibility temporarily limited medical service use. Similar disruptions have been reported for cancer, hypertension, and diabetes care in Korea and Japan [12,13,14,15], supporting the interpretation that the pandemic further accelerated an existing downward trajectory in CD utilization.

The expenditure trend identified in Table 1 was not statistically significant, indicating substantial inter-annual variability. The sharp increase in total expenditures observed in 2018 likely reflected factors beyond CD-specific care. The implementation of the “Moon Care” policy (2017–2018), which expanded insurance coverage for high-cost diagnostic tests such as brain and cervical MRI, as well as for inpatient services, substantially increased reimbursement costs. A descriptive post hoc review of expenditure trends across policy periods revealed temporal inflection points linked to major national events. Diagnostic testing expenditures rose sharply between 2017 and 2018, temporally coinciding with the Moon Care expansion of insurance coverage for MRI and inpatient services. In contrast, patient numbers and overall expenditures declined during 2019–2021, consistent with nationwide utilization drops during the COVID-19 pandemic, followed by partial recovery in 2022–2023. Although not formally tested, these descriptive patterns suggest that both policy-driven coverage expansion and pandemic-related access barriers contributed to the observed temporal variability. Additional contributors include the nationwide acceleration of per-capita medical spending [16] and demographic changes such as rapid population aging [17]. The subsequent rise in expenditures in 2023, despite only modest increases in patient numbers, is more plausibly explained by post-pandemic recovery, rising unit costs, and cumulative effects of aging, although these interpretations remain speculative without formal comparison between pre- and post-COVID periods. The divergent trajectories between Western Medicine (WM) and Korean Medicine (KM) warrant attention. While expenditure fluctuations were predominantly influenced by WM, KM utilization and costs remained low throughout the study period (Figure 2). This contrast likely reflects structural differences between the two systems—such as reimbursement incentives, patient preferences, and the fact that most KM treatments for cervical dystonia, including pharmacopuncture, are provided as non-covered (out-of-pocket) services.

Together, these findings suggest that changes in CD utilization are influenced by multiple interacting factors related to epidemiology, care pathways, and health system dynamics.

### 4.2. Demographic Characteristics

A female predominance in CD is well documented [18,19,20], with some multicenter data indicating earlier onset and higher patient counts in selected male subgroups [21]. Against this background, our findings reveal a sex–cost dissociation: women were more prevalent, but men generated greater expenditures in absolute terms, while women exhibited faster growth in the number of WM-treated patients and WM-related spending. However, regression analyses confirmed that the annual number of patients significantly declined among WM males and both KM sexes, whereas WM females showed no meaningful change. Expenditure trends, in contrast, did not reach statistical significance in any group, suggesting that observed sex-related cost gaps were driven primarily by differences in patient volume rather than per-patient intensity. Although CD-specific evidence on sex-based cost differentials is limited, similar trends have been reported in Parkinson’s disease, myocardial infarction, and advanced cancer care [22,23,24]. Possible mechanisms in CD include differences in case severity at presentation, thresholds for referral to resource-intensive services (e.g., inpatient care, imaging), treatment intensity once in WM pathways, and comorbidity profiles. Disentangling these factors will require procedure-level analyses (e.g., BoNT cycles, imaging episodes, admissions) and case-mix–adjusted models. Linking claims data with hospital registries or disease-severity indicators in future studies would help verify these hypotheses and move beyond speculation.

The divergent trajectories between WM and Korean Medicine (KM) also suggest system-level influences. Rising WM expenditures alongside declining KM patient numbers may reflect changes in reimbursement incentives and care pathways that favor WM; shifting patient preferences toward injection-based regimens; or provider-side dynamics such as availability and density. Given that overall expenditures did not change significantly, these patterns likely represent redistribution of patients across medical systems rather than cost escalation within a single modality. Generational effects could also play a role if younger cohorts increasingly rely on self-management or non-insured modalities not captured in claims data. Future work should link claims with provider-level indicators (e.g., geography, specialty mix) and survey patient-reported reasons for modality choice. Such divergence likely reflects reimbursement asymmetry within Korea’s dual medical system, suggesting that policy reforms expanding coverage for evidence-supported KM modalities could help balance care options and reduce long-term disparities in treatment accessibility.

Age-wise, patient distribution concentrated in middle-aged adults, consistent with the known epidemiology of adult-onset focal dystonia [25,26]. Linear-trend analyses further demonstrated that the number of patients decreased significantly in younger WM age groups (0–29 years), while remaining stable or increasing slightly in older populations. Increasing patient numbers among older adults (≥70 years) likely reflects Korea’s super-aged society and the burden of multimorbidity [17]. By contrast, growth in the 40–49 group may reflect earlier recognition, occupational impact prompting care-seeking, or wider adoption of guideline-concordant interventions. The significant decline among younger cohorts may indicate under-diagnosis of mild cases, pandemic-related disruptions, or substitution toward non-insured care such as physiotherapy or private-pay treatments.

These demographic trends align with publicly available, aggregated statistics from Korea’s Health Insurance Review & Assessment Service (HIRA Open Data portal) for 2018–2020, with further downturns during 2020–2022 attributable to pandemic-related access constraints [12,13,14,15]. Conversely, the rebound in 2023 aligns with system-wide recovery of services, rising unit costs, and aging-related pressure on healthcare spending. Overall, statistically significant decreases were confined to younger and KM-treated groups, highlighting that demographic aging and system-level treatment preferences jointly shape utilization patterns in cervical dystonia. These patterns are consistent with Andersen’s model, wherein predisposing characteristics (sex, age) and enabling factors (WM vs. KM access or reimbursement) differentially shape care-seeking behavior and intensity of healthcare use [27].

### 4.3. Evolving Cost Structure of CD Management

Service-specific expenditure demonstrated notable shifts. In WM, consultation costs remained relatively stable, while hospitalization expenditures declined modestly but without statistical significance. Neither was the major driver of overall growth. Instead, physiotherapy costs tended to increase consistently across inpatient and outpatient settings, underscoring the growing role of rehabilitation in long-term management. Evidence for physiotherapy as an adjunct to BoNT in CD is growing. Recent systematic reviews and meta-analyses report improvements in pain, disability, and quality of life with adjunctive physiotherapy, though samples remain modest and protocols heterogeneous; cost-effectiveness evidence is still limited and largely protocol-level to date [28]. Outpatient diagnostic testing also showed an upward trend (*p* < 0.001), while radiology declined, suggesting substitution or reimbursement changes.

Injection-related expenditures, including botulinum toxin (BoNT) therapy, rose slightly in inpatient care but declined significantly in outpatients (*p* = 0.01). Overall, BoNT expenditure remained stable, consistent with its status as the first-line therapy for CD, confirmed by randomized controlled trials, systematic reviews [29,30,31], and the 2015 international consensus guideline assigning Level A evidence [32]. Reimbursement stability likely explains the limited variation in BoNT-related costs.

In contrast, KM utilization generally contracted, but these decreases were not statistically significant, with most outpatient services declining and only limited inpatient growth. Although some small studies have suggested that acupuncture may offer benefits for patients with CD, such as reducing pain, improving motor function, and enhancing quality of life [33,34], the evidence remains preliminary. These limitations, together with barriers in access, awareness, or reimbursement, may explain why such potential benefits are not yet reflected in large-scale clinical practice. Similar analyses from Western countries have shown that botulinum toxin therapy accounts for most CD-related costs [7,8]. In Korea, however, the growing shares of diagnostic testing and rehabilitation expenditures indicate a different cost composition, likely reflecting system-level variations in service coverage and clinical practice.

### 4.4. Policy and Practice Implications

These patterns have several implications for clinical practice and health policy. The Korean expenditure structure—characterized by increasing diagnostic and rehabilitative costs rather than the dominance of botulinum toxin therapy observed in Western countries—suggests that service coverage and reimbursement frameworks substantially shape national spending priorities. In Western healthcare systems, where BoNT and specialist consultations account for the majority of expenditures, policy efforts typically emphasize maintaining access to advanced interventions. By contrast, Korea’s dual medical system distributes care across a broader spectrum of outpatient and supportive services, reflecting different reimbursement incentives and care delivery pathways. Future policy initiatives should ensure that reimbursement schemes, particularly for rehabilitation and evidence-supported Korean Medicine modalities, align with patient needs and long-term management goals. As utilization becomes increasingly concentrated in older adults, planning age-appropriate and multidisciplinary care will also be essential. These implications align with Andersen’s behavioral model, in which predisposing (sex, age) and enabling factors (coverage and reimbursement) shape healthcare utilization behaviors [27].

### 4.5. Specialty Distribution and Multidisciplinary Care in CD

Neurology accounted for the largest number of patients and visits, consistent with its role in diagnosis and botulinum toxin injections [35,36]. Neurosurgery, though smaller in patient volume, generated the highest expenditures and per-patient as well as per-visit costs, reflecting resource-intensive procedures such as selective peripheral denervation and deep brain stimulation [7,37,38].

Other frequently used specialties included anesthesiology and pain medicine, rehabilitation medicine, and orthopedic surgery. Rehabilitation was notable for both visit volume and expenditures, highlighting its supportive role in restoring function and managing pain [35,38,39]. KM specialties such as internal medicine and acupuncture also contributed with high visit frequency but low per-visit and moderate per-patient costs. Smaller contributions came from pediatrics, neuropsychiatry, and general surgery, underscoring the diversity of care pathways. Most neurological expenditures were associated with botulinum toxin injections, whereas neurosurgical costs were driven by DBS procedures; incorporating actual procedure counts in future analyses would strengthen clinical interpretability.

International multicenter surveys and patient-journey studies similarly emphasize the multispecialty and long-term nature of CD care, with patients transitioning across specialties depending on disease stage. These heterogeneous patterns highlight the importance of cross-specialty collaboration and integrated care models to optimize outcomes [36,40,41].

### 4.6. Strengths and Limitations

This study is the first nationwide analysis using the Korean National Health Insurance Service (NHIS) database to characterize real-world healthcare use and costs for cervical dystonia (CD) in Korea. Its descriptive design limits causal inference and prevents direct estimation of disease burden; observed trends do not indicate changes in prevalence, incidence, or severity. Clinical details such as symptom scales, functional outcomes, and treatment response were unavailable, precluding case-mix adjustment. Diagnostic or procedural codes may be misclassified, and some costs could include care for comorbidities. Although this study identified CD cases using the primary diagnosis code (G24.3) rather than a more restrictive case definition, this approach was considered appropriate for the study’s aim of describing nationwide healthcare utilization patterns rather than establishing precise epidemiologic incidence. Non-reimbursed services were also excluded—many Korean Medicine (KM) therapies, such as pharmacopuncture, are paid out-of-pocket—likely underestimating KM use and expenditures. Costs were converted to U.S. dollars at a fixed exchange rate without adjustments for inflation, purchasing power parity (PPP), or population size, which may exaggerate year-to-year variation. Overall, these findings reflect patterns within Korea’s insurance system and, while not fully generalizable to other settings, provide useful reference data for international comparison and policy evaluation.

## 5. Conclusions

This nationwide analysis with formally tested temporal trends of Korean NHIS claims demonstrates measurable shifts in the epidemiology and healthcare delivery of cervical dystonia (CD) from 2017 to 2023. Patient numbers declined significantly, particularly among younger groups and Korean Medicine users, while overall spending remained stable, reflecting redistribution rather than escalation of costs. Within Western Medicine, increases in diagnostic and rehabilitative services contrasted with numerical decreases in some procedure-related expenditures, suggesting a gradual transition toward supportive and evaluation-focused care. Collectively, these findings indicate that CD management in Korea is becoming more concentrated among older adults and multidisciplinary specialties, underpinned by stable use of botulinum toxin as the therapeutic mainstay. By quantifying these statistically significant demographic and service-level trends, this study provides a national benchmark for future monitoring, resource planning, and policy assessment in movement disorder care. While this study provides a robust national benchmark, its findings should be interpreted in light of inherent data limitations, such as the absence of clinical severity indicators and the exclusion of non-reimbursed services, which warrant further investigation in future research.

## Figures and Tables

**Table 1:** Trend analysis of annual changes in patient numbers and healthcare expenditures among patients with cervical dystonia, 2017–2023.

Outcome Variable	β (Annual Change)	*p*-Value	R^2^
Patients (Total)	−72.1	0.03	0.66
Expenditure (Total)	+16,917	0.36	0.17
Expenditure (WM)	+18,755	0.31	0.21
Expenditure (KM)	−1838	0.16	0.35

WM = Western Medicine; KM = Korean Medicine; β = regression coefficient for year (2017–2023). Linear regression models were used with year as a continuous variable. A *p*-value < 0.05 was considered statistically significant.

**Table 2:** Annual Healthcare Utilization and Temporal Trends (2017–2023) by Treatment Type and Sex.

Treat	Sex	Avg. No. of Patients per Year	Avg. Annual Exp (USD)	Avg. per Visit (USD)	Avg. per Pt (USD)	CAGR Exp	CAGR Pt	β Exp (95% CI)	*p*	β Pt (95% CI)	*p*
WM	Female	599	139,000	71	229	15.37	0.53	20,274 (−6534–47,106)	0.11	−9.46 (−46.85–27.92)	0.54
	Male	507	165,000	97	330	10.88	−6.23	−1533 (−29,974–26,915)	0.9	−39.25 (−54.42–−24.08)	0
KM	Female	100	13,580	20	140	−7.75	−12.71	−1024 (−2618–570)	0.16	−12.71 (−22.26–−3.17)	0.02
	Male	72	11,270	23	165	−6.43	−14.11	−814 (−2122–494)	0.17	−10.64 (−18.85–−2.43)	0.02

WM, Western Medicine; KM, Korean Medicine. Avg. No. of Patients per Year represents the mean number of unique patients per year from 2017 to 2023. Avg. per Visit and Avg. per Pt denote the average medical expenditure per visit and per patient, respectively. CAGR Exp and CAGR Pt indicate the compound annual growth rates of total healthcare expenditure and the number of patients. β Exp and β Pt represent the annual changes in total expenditure and patient numbers, respectively, estimated using simple linear regression models. All expenditures are presented in U.S. dollars, converted from Korean won at an exchange rate of 1 USD = 1388 KRW.

**Table 3:** Annual Healthcare Utilization and Temporal Trends (2017–2023) by Treatment Type and Age.

Treat	Age	Avg. No. of Patients per Year	Avg. Annual Exp (USD)	Avg. per Visit (USD)	Avg. per Pt (USD)	CAGR Exp	CAGR Pt	β Exp (95% CI)	*p*	β Pt (95% CI)	*p*
WM	0–9	216	7549	17	35	−7.41	−8.10	−665 (−1329~0)	0.05	−21 (−30~−13)	0.00
	10–19	59	5718	57	88	−13.32	−8.10	−1990 (−59,934~2015)	0.26	−6 (−9~−3)	0.01
	20–29	53	8373	63	152	−13.99	−7.41	−1531 (−3031~−30)	0.05	−5 (−8~−2)	0.01
	30–39	84	25,534	91	308	0.33	−3.22	−803 (−3468~1860)	0.47	−4 (−10~1)	0.10
	40–49	143	49,320	105	354	31.88	−4.62	6589 (−3602~16,784)	0.16	−8 (−13~−3)	0.01
	50–59	194	87,266	111	455	4.5	−1.59	353 (−7019~7728)	0.91	−6 (−14~2)	0.10
	60–69	194	79,164	103	396	19.08	6.54	10,630 (−6527~27,780)	0.17	11 (−5~27)	0.14
	70–79	120	35,384	79	300	31.57	−3.20	5633 (−4689~15,950)	0.22	−8 (−24~8)	0.25
	≥80	40	5792	35	141	20.05	0.00	542 (−884~1969)	0.37	−1 (−3~2)	0.48
KM	0–9	1	44	15	27	NA	NA	−10 (−34~13)	0.32	−1 (−1~0)	0.07
	10–19	3	112	19	30	−28.55	−23.53	−46 (−80~−11)	0.02	−1 (−2~0)	0.02
	20–29	12	2055	24	143	−41.97	−26.32	−829 (−1363~−294)	0.01	−3 (−5~−1)	0.00
	30–39	19	3239	24	267	−4.61	−16.05	167 (−520~854)	0.56	−5 (−8~−1)	0.01
	40–49	30	4887	28	164	−15.28	−21.75	−933 (−2058~194)	0.09	−6 (−11~−2)	0.02
	50–59	43	5169	21	120	−5.02	−11.37	−574 (−1608~460)	0.21	−5 (−10~1)	0.08
	60–69	36	6561	21	184	−6.42	−8.46	−47 (−1081~988)	0.91	−3 (−6~0)	0.05
	70–79	21	2517	16	127	21.19	−6.00	385 (−237~1007)	0.17	−1 (−3~2)	0.59
	≥80	5	273	15	55	23	8.15	47 (−20~114)	0.13	0 (−0~1)	0.45

WM, Western Medicine; KM, Korean Medicine. Avg. No. of Patients per Year represents the mean number of unique patients per year from 2017 to 2023. Avg. per Visit and Avg. per Pt denote the average medical expenditure per visit and per patient, respectively. CAGR Exp and CAGR Pt indicate the compound annual growth rates of total healthcare expenditure and the number of patients. β Exp and β Pt represent the annual changes in total expenditure and patient numbers, respectively, estimated using simple linear regression models. All expenditures are presented in U.S. dollars, converted from Korean won at an exchange rate of 1 USD = 1388 KRW.

**Table 4:** Expenditures and Compound Annual Growth Rates by Service Category.

	WM Inpatient			WM Outpatient			KM Inpatient			KM Outpatient		
	Avg. No. of Visits	CAGR (%)	*p*	Avg. No. of Visits	CAGR (%)	*p*	Avg. No. of Visits	CAGR (%)	*p*	Avg. No. of Visits	CAGR (%)	*p*
Consultation fee	53	1.6	0.14	3521	−0.3	0.09	2	23.2	0.41	1216	−12.2	0.73
Hospitalization fee	51	−2.9	0.76	-	-	-	3	−8.2	0.43	-	-	-
Medication fee	46	1.1	0.21	6	−9.1	0.96	1	-	1.00	76	16.2	0.12
Injection fee	41	2.9	0.16	247	−5	0.01	-	-	-	-	-	-
Anesthesia fee	20	5.9	0.11	659	−13.2	0.01	-	-	-	-	-	-
Physiotherapy fee	35	8.9	0.10	1420	6.6	0.58	-	-	-	-	-	-
Psychotherapy fee	1	−12.9	0.47	4	-	0.03	-	-	-	-	-	-
Treatment fee	22	0	0.61	18	−10.2	0.63	3	5.8	0.99	1208	−12.1	0.53
Testing fee	48	1.7	0.34	147	11.1	<0.001	1	-	0.19	11	−40.5	0.74
Radiology fee	44	0.7	0.20	271	−12	0.61	-	-	-	-	-	-

KM, Korean Medicine; WM, Western Medicine. Avg. No. of Visits indicates the annual average number of claims per service category (2017–2023). CAGR (%) represents the compound annual growth rate in visit counts. Trend *p*-values were obtained from log–linear Poisson regressions of annual visit counts on calendar year, with log(no. of persons) as an offset; models with overdispersion were fit using negative binomial or quasi-Poisson as appropriate.

**Table 5:** Top 10 Specialties Visited by Patients with Cervical Dystonia.

Specialty	Patients (n)	Visits (n)	Total Expenditure	Avg. per Visit	Avg. per Pt
			(USD)	(USD)	(USD)
Neurology	2981	10,282	504,908	49	169
Anesthesiology & Pain Medicine	874	2741	110,414	40	126
Internal Medicine (KM)	616	3842	77,026	20	125
Orthopedic Surgery	558	1788	42,189	24	76
Neurosurgery	554	6434	1,295,531	201	2338
Rehabilitation Medicine	344	2815	117,910	42	342
Pediatrics & Adolescents	259	404	9060	22	35
Neuropsychiatry (KM)	206	1327	23,401	18	114
General Surgery	182	419	27,796	66	153
Acupuncture (KM)	121	3082	61,757	20	510

Avg. per Visit was calculated as total expenditure divided by the number of visits, and Avg. per Patient as total expenditure divided by the number of patients. Costs were converted to U.S. dollars (USD) using the rate of 1 USD = 1388 KRW.

## Data Availability

The data that support the findings of this study are available from the Korean National Health Insurance Service (NHIS). Restrictions apply to the availability of these data, which were used under license for the current study. Data are available from the NHIS upon reasonable request and with permission of the NHIS (https://nhiss.nhis.or.kr/) (accessed on 17 November 2025).

## Supplementary Materials

The following supporting information can be downloaded at: https://www.mdpi.com/article/10.3390/healthcare13222995/s1, Table S1: Healthcare Utilization among Patients with Cervical Dystonia in Korea, 2017–2023.

## Abbreviations

The following abbreviations are used in this manuscript:

BoNT	Botulinum toxin
CAGR	Compound annual growth rate
CD	Cervical dystonia
HIRA	Health Insurance Review and Assessment Service
KM	Korean Medicine
WM	Western medicine
