# Supplementary material for: Nationwide Trends in Healthcare Utilization and Expenditures Among Patients with Cervical Dystonia in Korea: A 7-Year Analysis Using Health Insurance Data
Source: Healthcare (Basel). 2025 Nov 20;13(22):2995. doi: 10.3390/healthcare13222995 (PMC12652393; doi:10.3390/healthcare13222995)
Supplement: Supplementary file 1 [file healthcare-13-02995-s001.zip › healthcare-3925123-supplementary.pdf]

Supplementary Table S1. Healthcare Utilization among Patients with Cervical Dystonia in Korea, 2017–2023

Year	Type of Visit	Number of Patients	Total Claims	Total Expenditure (USD)
2017	Total	1,589	5,890	257,923
	WM	1,297	3,869	221,393
	KM	292	2,021	36,530
2018	Total	1,464	5,420	396,757
	WM	1,284	3,855	368,847
	KM	180	1,565	27,911
2019	Total	1,318	5,337	324,993
	WM	1,124	3,969	298,889
	KM	194	1,368	26,105
2020	Total	1,141	4,111	260,535
	WM	1,012	3,329	245,067
	KM	129	782	15,468
2021	Total	1,098	4,215	253,455
	WM	940	3,273	234,191
	KM	158	942	19,264
2022	Total	1,093	4,134	313,962
	WM	970	3,210	288,695
	KM	123	924	25,267
2023	Total	1,237	4,789	494,855
	WM	1,113	3,858	471,436
	KM	124	931	23,419
